# Elucidation of the conformational free energy landscape in *H.pylori *LuxS and its implications to catalysis

**DOI:** 10.1186/1472-6807-10-27

**Published:** 2010-08-12

**Authors:** Moitrayee Bhattacharyya, Saraswathi Vishveshwara

**Affiliations:** 1 Molecular Biophysics Unit, Indian Institute of Science, Bangalore-560012, India

## Abstract

**Background:**

One of the major challenges in understanding enzyme catalysis is to identify the different conformations and their populations at detailed molecular level in response to ligand binding/environment. A detail description of the ligand induced conformational changes provides meaningful insights into the mechanism of action of enzymes and thus its function.

**Results:**

In this study, we have explored the ligand induced conformational changes in *H.pylori *LuxS and the associated mechanistic features. LuxS, a dimeric protein, produces the precursor (4,5-dihydroxy-2,3-pentanedione) for autoinducer-2 production which is a signalling molecule for bacterial quorum sensing. We have performed molecular dynamics simulations on *H.pylori *LuxS in its various ligand bound forms and analyzed the simulation trajectories using various techniques including the structure network analysis, free energy evaluation and water dynamics at the active site. The results bring out the mechanistic details such as co-operativity and asymmetry between the two subunits, subtle changes in the conformation as a response to the binding of active and inactive forms of ligands and the population distribution of different conformations in equilibrium. These investigations have enabled us to probe the free energy landscape and identify the corresponding conformations in terms of network parameters. In addition, we have also elucidated the variations in the dynamics of water co-ordination to the Zn^2+ ^ion in LuxS and its relation to the rigidity at the active sites.

**Conclusions:**

In this article, we provide details of a novel method for the identification of conformational changes in the different ligand bound states of the protein, evaluation of ligand-induced free energy changes and the biological relevance of our results in the context of LuxS structure-function. The methodology outlined here is highly generalized to illuminate the linkage between structure and function in any protein of known structure.

## Background

Enzymes perform their function by responding precisely to their cognate ligands, yet retaining their overall shape and structure. This phenomenon was beautifully formulated in classical models such as the "MWC" model of Monod [1] and the "KNF" model of Koshland [2]. Such a phenomenological model, known as allostery, has played a major role in biochemistry in explaining the functioning of enzymes. At a quantitative level however, the effect of ligand binding on the enzyme can be subtle, multifold, ranging from minor local conformational change to major global changes or altering the conformational populations, which can affect the conformational free energy [3-5]. The pre-existence of an ensemble of pathways for allosteric communication has also been proposed [6]. The concept of catalytic free energy landscape in the context of the function of enzymes has been discussed in literature [7-9]. A quantitative approach to elucidate such a landscape has been taken by Warshel's group [9-12]. The correlation of events such as protein conformational mobility and enzyme action through similar timescales has been an approach to elucidate the landscape [7,8,13]. Based on rigorous theoretical studies, it has been suggested that enzyme fidelity is determined by the proper pre-organization in the enzyme-substrate (ES) form [12] and catalysis is guided by the barrier to the chemical step in the ES complex and not affected by induced fit [10,11] and these concepts have been extensively reviewed in [9]. At the basic level, it is valuable to capture the conformational changes from a detailed molecular perspective. The structures of proteins bound to different ligands obtained from X-ray and NMR studies greatly enhance our understanding of the ligand-induced changes [14,15]. The challenge has been to capture the subtle conformational changes and obtain the conformational populations in a precise manner that can help to construct a free energy landscape [8]. Graph theoretical approaches have proved to be useful in capturing the side-chain interactions from a global perspective through the representation of protein structures in the form of protein structure networks (PSN) [16-18]. The importance of minor side-chain variations in the light of 'allostery' has also been established [4,19]. Our previous studies on tRNA synthetases [20,21] have shown that a combination of molecular dynamics simulations and protein structure network analysis effectively captures the allosteric paths of communication. It has also been suggested that the allosteric energy coupling in enzymes requires the consideration of the coupling with transition state energy [22]. Furthermore, our recent studies have shown that the conformational changes described by the network of side-chain interactions and redistribution of conformational populations can be accurately mapped onto free energy changes [23].

In the present study, we have investigated the structure-function relationship in LuxS by performing MD simulations on the homodimeric protein LuxS from *H.pylori *[24] and its different ligand bound structures, and by characterizing the ensemble of conformations through protein structure network analysis. We have also obtained the network of residues around the active sites, in the different ligand bound states of LuxS. Specifically we have identified ligand induced changes in conformation and free energy in LuxS and its different ligand bound forms using dynamically stable network parameters and essential dynamics approach in unison. Additionally we also demonstrate that the ligand-induced changes in the network of amino acid interactions in the enzyme can influence catalytic mechanisms.

LuxS is a bacterial protein, implicated in quorum sensing, which enables cross-talk across the bacterial 'societies' to bring about efficient synchronization of action as a function of cell density [25-27]. The specific signalling molecules involved are autoinducer 1 and 2 [26] (abbreviated as AI-1 and AI-2 respectively). LuxS is involved in the biosynthetic pathway of AI-2, the universal signal for bacterial inter-species communication. In some organisms, quorum sensing by LuxS imparts a profound effect on pathogenicity by affecting toxin production/flagellar morphogenesis and hence motility and colonization. However, in some other species no direct pathogenic role has been assigned and LuxS is known to affect the metabolic processes [25]. LuxS cleaves the thioether bond in S-Ribosylhomocysteine to produce homocysteine and DPD, the precursor molecule for AI-2 production in the S-adenosylmethionine (SAM) cycle [28]. Such reactions are usually catalyzed by enzymes that require a redox-active cofactor. However, LuxS does not require such cofactors to catalyze this chemically difficult reaction [28]. Previously we have probed into the structure-function relationships in LuxS (both crystallographic and modelled structures) from several bacterial species by employing the method of graph theory [29]. In the present study, we have investigated ligand induced conformational changes in *H.pylori *LuxS, which provides important structural details and mechanistic insights into the catalytic cycle of this dimeric protein. It is an interesting system to target for development of broad-spectrum antibiotics as no LuxS homologues are present in the human. So its detailed investigation becomes exceedingly important.

## Methods

### Molecular Dynamics Simulations

Molecular dynamics simulations are performed at 300 K using AMBER9 [30] with parm99 parameters [31] on five *H.pylori *LuxS systems, namely LuxS_apo (PDB: 1J6X), LuxS+SRH, LuxS+2SRH, LuxS+KRI, and LuxS+2KRI. The five simulations are carried out for 10 ns each in aqueous medium using TIP3P water model. The solvation box is 10Å from the farthest atom along any axis. The simulations are performed under NPT conditions. The van der Waals cutoff used is 10Å and the pressure and temperature relaxations are set to 0.5 ps^-1^. A time step of 2 fs is employed with the integration algorithm and the structures are stored every 1 ps.

### Modeling of the ligand (SRH/KRI) bound structures from H.pylori

The crystal structure of *H.pylori *LuxS has been solved [32]. However structures of *H.pylori *LuxS bound to its substrate S-Ribosylhomocysteine with intact ribose ring (SRH) or the 2-ketone intermediate (KRI) is not available. So we use the SRH and KRI bound LuxS structures from *B.subtilis *[PDB: 1JVI[33] and 1YCL[34] respectively] as a template to model the corresponding structures from *H.pylori *(LuxS+SRH-LuxS+2KRI). The orientation of the *H.pylori *LuxS which is suitable for complex formation with SRH/KRI is generated by superposing *H.pylori *LuxS on the corresponding ligand bound structures of *B.subtilis *LuxS (backbone rmsd is 0.742, all-atom rmsd is 0.644). The LuxS from *H.pylori *and *B.subtilis *has high sequence similarity (Score = 134, e-value = 2e^-32^) and identity of 46.6%. We further validated our docking by inspection of side-chain interactions involving the residues forming the active sites of LuxS in the two species. The communities (described in detail in the sub-section ***Network parameters associated with high connections) ***involving the histidines and cysteine forming the active site of LuxS has same/similar residues showing the similarities in the two ligand binding pockets [Additional file 1: Supplemental Figure SA1(a)]. We pictorially inspected the status of the ligand and the binding pocket after docking in *H.pylori *LuxS with reference to *B.subtilis *LuxS and an almost identical position is observed for the active site residues in the two species validating our docking exercise [Additional file 1: Supplemental Figure SA1(b)].

### Construction of Protein Structure Network

Protein Structure Network/Protein Structure Graph (PSN/PSG) effectively captures the non-covalent side-chain interactions from a global perspective. The details of the construction of such a graph at a particular interaction cut-off (I_min_) and the implications of such graphs have been discussed in detail previously[35,36]. A brief review is presented here. Protein structure networks are constructed by considering amino acid residues as *nodes *and *edges *are constructed between the nodes on the basis of non-covalent interactions between them (as evaluated from the normalized number of contacts between them) for each system. The non-covalent interaction between side chain atoms of amino acid residues (with the exception of Gly where C_α _atom) are considered, ignoring the interaction between sequence neighbours. The interaction between two residues i and j has been quantified previously in our lab as:

$${\text{l}}_{\text{i}\text{j}}=\frac{{\text{n}}_{\text{i}\text{j}}}{\sqrt{\left({\text{N}}_{\text{i}}\times {\text{N}}_{\text{j}}\right)}}\;\times \;100$$

where n_ij _is number of distinct atom pairs between the side chains of amino acid residues i and j, which come within a distance of 4.5Å and N_i _and N_j _are the normalization factors for residues i and j[35]. The pair of amino acid residues having interaction strength (I_ij_) greater than a user-defined cut-off (I_min_) are connected by edges to give a protein structure network (PSN) graph for a given interaction strength I_min_. Generally, I_min_s in the PSNs vary from 1% to 15%. The lower the I_min_, the higher is the connectivity. An analysis of a large number of protein structures had shown that the optimal interaction strength in a protein structure is exhibited at the point the size of the largest noncovalently connected cluster undergoes a transition [16]. This I_min _which is around 2-4% for most of the protein structures (including the present system) is termed I_critical. _The number of residue-residue interactions drastically reduces above this I_min_. In the present study, an I_min _of 2.5% is used for the evaluation of network parameters. Any pair of residues is considered to be connected if I_min _is greater than 2.5%.

### Dynamically Stable Hydrogen Bonds

The hydrogen bonds within the protein and between protein-ligand are generally identified in the structures obtained from X-ray crystallography. Although it is a reasonably accurate representation, some changes can be expected to take place in the solution environment. The average behaviour in the aqueous environment can be captured from the equilibrium dynamics simulations. Specifically, the hydrogen bonds (both mainchain-mainchain and mainchain-sidechain) are computed over the simulation trajectory and those present in > 50% of the snapshots are termed as dynamically stable ones and used for the analysis. The focus of this analysis here is mainly on the interactions between the ligands (SRH/KRI) and the protein.

### Dynamic Cross-Correlation Maps

Dynamic Cross-correlation maps are used to identify the regions that move in or out of phase during the simulations. The construction and significance of such maps has been described in detail elsewhere[20]. The cross-correlation coefficient varies from-1 (completely anti-correlated motion) to +1 (completely correlated motion).

In this study, the cross-correlation coefficients are computed by considering the backbone Cα atoms of amino acids in the protein using PTRAJ module in AMBER9 [37]. The ensemble average structure is obtained from the snapshots from 0.5-10 ns.

### Interface Dynamics Analysis

Protein-protein interactions generally take place through non-covalent interactions and this feature is also sensitive to environment such as the medium and the complex state of the protein. Graph theory based cluster analysis of the structures has been shown to elucidate the different types of interfaces in multimeric proteins such as lectins [17] and tryptophanyl-tRNA synthetase [38]. In the present study of the dimeric protein LuxS, the interface clusters present in the snapshots from the simulations are identified (using the algorithm *Depth First Search*). The identified interface clusters are held together by non-covalent interactions. It has been previously shown that the clusters identified at the interaction strength I_min _≥ 6% play a significant role in stabilizing the interface [18]. Hence we have used an I_min _of 6% for the identification of interface clusters from the simulation snapshots.

### Network parameters associated with high connections

In general network terminology, the parameters cliques/communities represent tightly connected regions of the network [39] and hubs represent highly connected points. In the context of PSN, these parameters are used to identify the rigid regions in the protein structures and to recognize the changes that take place due to the binding of ligands [21]. In this study, the PSNs are constructed as described above for all the snapshots of the five MD simulated structures. We analyze these PSNs for k-clique communities and hubs on the basis of the definitions that follow.

#### k-clique community

A k-clique is defined as a set of k nodes (points represented by amino acids) in which each node is connected to all the other nodes. A community is defined as a union of smaller k-cliques that share node/s. According to mathematical literature, a k-clique community has been defined as the assemblage of k-cliques that can be percolated through a series of adjacent k-cliques. In the present study we have used a variation of the community definition and a k-clique community is one in which two k-cliques share k-1 or k-2 nodes. Community size is determined by the number of constituent cliques and is considered to be proportional to the compactness or packing density in the proteins.

#### k-Clique community finding algorithm

The community search approach employed by us is based on the algorithm proposed by Palla *et al *[39]. We have used Cfinder[40] to obtain the k-clique community from PSNs. In majority of the cases we obtain k = 3 cliques (with a few exception of k = 4 cliques) at the chosen I_min _= 2.5%. k-clique communities with an overlap of k-1 nodes are obtained using Cfinder. The communities with k-2 node overlaps are obtained by manual inspection of the cliques and communities.

#### Hubs

At a certain I_min _different nodes have different number of connectivity (i.e. edges) with its neighbours. Hubs are defined as nodes connected by four or more edges to its neighbours in the PSN at the chosen value of I_min_.

#### Dynamically Stable Cliques, Communities, and Hubs

The network parameters are considered to be dynamically stable if they are present in more than 50% of the simulation snapshots. The dynamically stable ones represent the major conformational states in the MD ensemble. The two-dimensional representation of these dynamically stable cliques and communities are drawn using Cfinder.

### Essential Dynamics, Conformational Re-distribution, and Helmholtz Free Energy

Ideally the complete enumeration of the "catalytic free energy landscape" involves rigorous procedures taking into consideration the chemical barrier along the reaction coordinate [10-12]. Our present study however is limited to the investigations of the ligand induced variations in the protein conformations and the changes in the Helmholtz free energy of each system. We thus calculate discreet state information about definite points on the landscape. This involves the analysis of simulated trajectories in terms of essential dynamics and the method is briefly summarized below.

Essential dynamics is a robust technique for the identification of the 'essential subspace' from the protein dynamics representing the major conformational degrees of freedom for a protein [41]. For each of our five simulations, all the snapshots obtained from the MD trajectory during the production run are superposed with respect to the average MD structures using all Cα atoms. A covariance matrix of positional fluctuation is then calculated using the positional coordinates of all the heavy atoms from equation 1.

(1) $$\text{Mij}=\left(\text{1}/\text{S}\right)\sum_{\text{t}}\left({\text{x}}_{\text{i}}(\text{t})-\;<{\text{x}}_{\text{i}}>)({\text{x}}_{\text{j}}(\text{t})-\;<{\text{x}}_{\text{j}}>\right)$$

Where, S is the total number of snapshots, t is time in ps, x_i _is the i^th ^coordinate (xi = 1, 2, ...., 3N; N being the number of Cα atoms). The covariance matrix is then diagonalized to obtain eigenvalues and eigenvectors using MATLAB and the relative cumulative positional fluctuation (RCPF) is evaluated for each system using equation 2.

(2) $$RCPF\left(n\right)=\frac{\sum_{i=1,n}\lambda \left(i\right)}{\sum_{i=1,3N}\lambda \left(i\right)}$$

Where λ(i) is the i^th ^eigenvalue and RCPF(n) is amount of motion associated with the subspace spanned by the first n eigenvalues. Sorting the eigenvalues in the descending order enables segregation of the conformational space in a low dimensional (essential subspace) subspace to which the major conformational fluctuations are restricted. It has been shown in previous studies that relevant conformational transitions are not detectable on other planes, and the corresponding eigenvectors are thus not suitable as conformational coordinates to describe the large conformational fluctuations [41,42]. In our study, we have considered the two principal components with the highest eigenvalues to define the 'essential plane' for our analysis.

The 'essential plane' is then divided into m^2 ^cells using m × m grids. The conformations sampled every 1 ps is then projected on to this 'essential plane' and a binning of the conformations in these cells is done and plotted to obtain the population distribution profile using MATLAB. For each cell, relative probabilities are calculated with reference to the cell containing maximum number of points for calculation of Helmholtz free energy using equation 3[43,44].

(3) $$\Delta A_{ref\to i}=\;-RT\ln\frac{p_{i}}{p_{ref}}$$

Where R is ideal gas constant, T is temperature (300K), and p_i _and p_ref _are the probabilities of finding the system in i^th ^cell and the reference cell respectively. The minimum Helmholtz free energies in a contour map are also calculated using equation 4.

(4) $${\text{A}}_{\text{i}}={\text{A}}_{\text{ref}}-\text{RTln}\left({\text{p}}_{\text{i}}/{\text{p}}_{\text{ref}}\right)$$

where A_ref _is the free energy of our reference cell, A_ref _= -RTln(p_ref_/p_TOT_) and p_TOT _is the total number of conformations.

## Results

MD Simulations are performed on *H.pylori *LuxS (LuxS_apo) and its four ligand bound (S-ribosylhomocysteine in both the active (KRI) and inactive (SRH) forms bound to one or both the subunits) forms (LuxS+SRH - LuxS+2KRI) (10ns each) to obtain equilibrated structures which are analyzed as described in the subsequent sections.

### Root Mean Square Deviation (RMSD) Profiles

**Figure 1:**
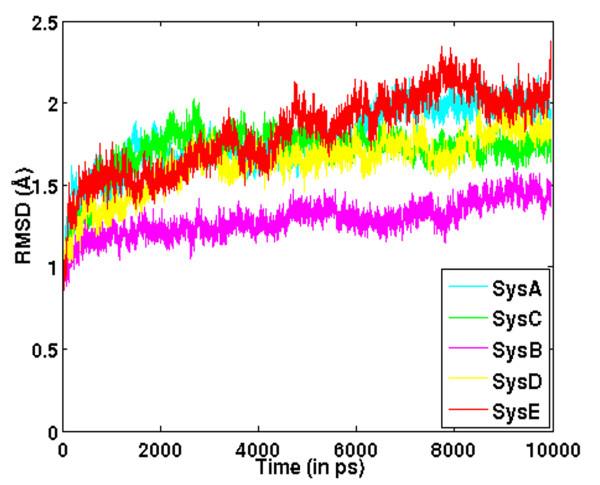
**RMSD profiles of LuxS in different ligand bound states**. Root-mean-square deviation (RMSD) profiles of LuxS in its various states of ligation [LuxS_apo, LuxS+SRH, LuxS+2SRH, LuxS+KRI, LuxS+2KRI] with reference to the minimized crystal structures.

It is evident from the backbone RMSD profile [Figure 1] that the trajectories do not differ from each other drastically till the end of simulation time (10 ns). However, the RMSD profiles of the five systems exhibit subtle differences (mainly between 6-10 ns), indicating the occurrence of some minor conformational variations in this time range, which would also be validated by our subsequent analyses.

### Residue-wise RMSD (RRMSD) Profiles

In order to probe further into the regions contributing to the overall backbone RMSDs, the simulation averaged RRMSD values are evaluated for LuxS_apo-LuxS+2KRI [Additional file 1: Supplemental Figure SA2]. The RRMS deviations are maximum in LuxS_apo. The asymmetry between the two subunits is hinted at by Additional file 1: Supplemental Figure SA2 with residues from subunit A/B of a system fluctuating to different extents. Although such an analysis gives an idea of average deviation per residue over the trajectory for a particular system, it gives no clue to the correlated fluctuations of residues in the different systems in atomistic detail, an aspect, which is addressed in a later section.

### Interactions at the Active Site

**Figure 2:**
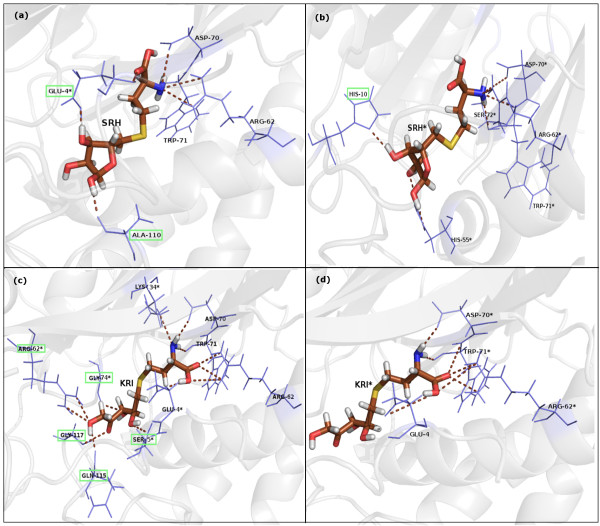
**Dynamically stable hydrogen bonds between protein and ligand**. Dynamically stable hydrogen bonds (% of occurrence > 50) for LuxS+2SRH (a) Subunit A and (b) Subunit B and for LuxS+2KRI (c) Subunit A and (d) Subunit B between the protein and ligand. The hydrogen bonds are marked with brown dashed lines. The ligand is depicted in stick representation and the protein residues are represented as slate blue lines. The asymmetry in hydrogen bonding pattern in the two subunits of LuxS+2SRH and LuxS+2KRI around the ribosyl moiety of ligand (SRH/KRI) is highlighted with green rectangular boxes.

Dynamically stable hydrogen bonds (present in ≥ 50% of the snapshots) made by the ligand (both inactive (SRH) and active (KRI) ligand) with LuxS residues are listed in Additional file 2: Supplemental Table SB1(a-b) for LuxS+KRI and LuxS+2KRI (active form) and Additional file 2: Supplemental Table SB2(a-b) for LuxS+SRH and LuxS+2SRH (inactive form) respectively. A pictorial representation of dynamically stable hydrogen bonds (H-bonds) between ligand and LuxS residues for the two subunits of LuxS+2SRH and LuxS+2KRI are also given in Figure 2(a-d) respectively. A marked increase in the number of dynamically stable H-bonds (Mainchain-Sidechain) around the ligand in each subunit of the bi-liganded structures (LuxS+2SRH and LuxS+2KRI) as compared to the mono-liganded structures (LuxS+SRH and LuxS+KRI) signifies increased compactness and rigidity around the active sites in the biliganded enzyme.

An important point to note is the asymmetric nature of the H-bonding pattern in the two subunits of LuxS in LuxS+2SRH and LuxS+2KRI [Figure 2(a-d)] inspite of the prediction about the homodimeric nature of the protein from its crystal structure (PDB_id:1J6X). The H-bonding pattern is highly symmetric as far as the homocysteine moiety of the ligands (SRH/KRI) is concerned. However, the asymmetry is marked in the nature of H-bonds between the ribosyl moiety (both in the open and closed forms) of the ligand and LuxS residues as highlighted in Figure 2(a-d). Particularly, in the 2-ketone intermediate bound form, LuxS+2KRI, the difference in the pattern of H-bonding around KRI in the two subunits is strikingly asymmetric. This asymmetry is further probed in our subsequent analysis of the simulation trajectories.

### Dynamic Cross-Correlations

**Figure 3:**
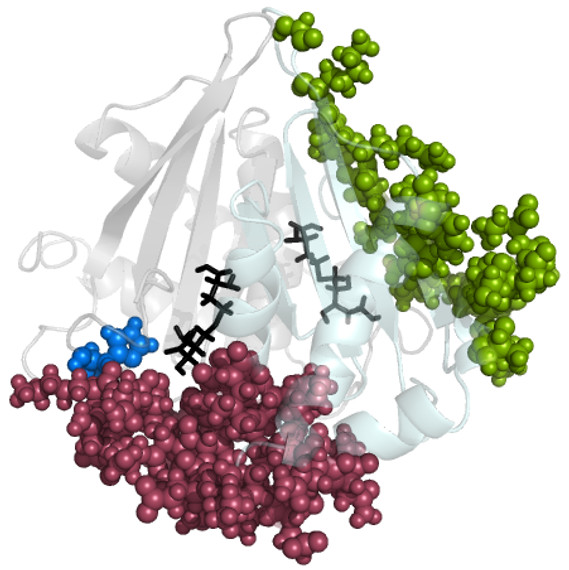
**Pictorial representation of the inter subunit correlated and anti-correlated residues of 10 H in LuxS+2KRI**. The backbone is represented as cartoon with the two subunits being coloured differently. The correlated and anti-correlated residues are given as maroon and green van der Waals spheres. 10 H is represented as a blue van der Waals sphere. The ligand is given in black sticks representation.

Cross-correlation coefficients are computed from the MD trajectories as described in the Methods section. The dynamic cross-correlation maps (DCCM) have been generated for the five systems, LuxS_apo-LuxS+2KRI [Additional file 1: Supplemental Figure SA3(a-e)]. The comparison between the DCCMs obtained from LuxS_apo-LuxS+2KRI clearly indicates that the binding of ligands to LuxS induce variations in correlated fluctuations between the two subunits of LuxS to different extents. A large number of correlated fluctuations appear in LuxS_apo because of the lack of rigidity. The biliganded structures which are maximally rigid have decreased correlated fluctuations. A distinct pattern is seen for the active and inactive ligand bound forms of the enzyme with the appearance of the inter subunit anti-correlations in the active ligand bound complexes. This clearly indicates that different ligands influence inter subunit cross-talk to different extents. It is interesting to note that some of the residues [H10Q, R38 M, E54Q, K40A, and C75A] from the correlated pairs have been shown to be important from mutation experiments [45]. The inter subunit correlations and anti-correlations with one of these residues (10H) is pictorially depicted in Figure 3. The correlated residues are majorly near the active sites/interface whereas the anti-correlated residues are away from the interface. It is to be noted that there is a prominent variation in correlation involving the residues drastically affecting catalytic activity in one subunit and the active site (and its nearby residues) from the other subunit of LuxS.

### Study of Interface Dynamics using Cluster Analysis

In our analysis, dynamically stable interface residues are defined as those that have above 35% participation in the interface clusters from either subunit. The analysis clearly manifests that the subunits in LuxS are asymmetric in nature in terms of the dynamically stable residues participating to the interface clusters [an exhaustive description of such residues and their participation to the interface for LuxS_apo-LuxS+2KRI is pictorially depicted in Additional file 1: Supplemental Figure SA4(a-e)]. To further investigate this point, we evaluate the number of dynamically stable residues that have a minimum of 15%, 25%, and 35% difference in their participation to the dimer interface from the two subunits and the results are presented in Table 1. An inspection of Additional file 1: Supplemental Figure SA4(a-e) and Table 1 brings out the inherent asymmetry of native LuxS which is further enhanced to different extents upon ligand binding.

**Table 1:** Asymmetry associated with dynamically stable interface residues between the two subunits of LuxS_apo-LuxS+2KRI

Difference in participation between residues from the two subunits (in %)	No. of residues for LuxS_apo	No. of residues for LuxS+SRH	No. of residues for LuxS+2SRH	No. of residues for LuxS+KRI	No. of residues for LuxS+2KRI
15	22	34	27	32	26

25	14	20	18	23	25

35	7	16	8	18	13

### Network Parameters

The trajectories obtained from the five simulations are used to construct protein structure networks for each snapshot, which are then analyzed to probe into the dynamical properties of the network. Cliques, communities and hubs form the rigid regions in a protein structure network (PSN), and the dynamically stable ones correspond to the major conformations in the MD ensemble. We have analysed all these parameters from the simulations of LuxS_apo-LuxS+2KRI as described below.

### Cliques and Communities

**Figure 4:**
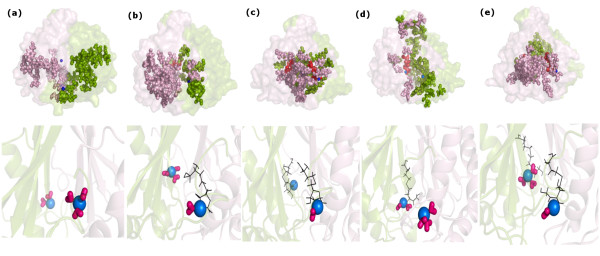
**Pictorial representation of the major cliques/communities and water coordination around Zn^2+^**. Pictorial representation of the major cliques and communities in (a) LuxS_apo, (b) LuxS+SRH, (c) LuxS+2SRH, (d) LuxS+KRI, and (e) LuxS+2KRI (upper panels). For LuxS_apo, LuxS+SRH, and LuxS+KRI all communities are plotted whereas for LuxS+2SRH and LuxS+2KRI, only the giant communities around the active sites are plotted for the sake of clarity [upper panel]. The two different subunits are represented as transparent surface and are coloured differently. The clique and community residues are represented as van der Waals spheres and coloured according to the subunit to which they belong. The ligand and Zn^2+ ^are also given in van der Waals representation and coloured red and blue respectively. In the lower panel, water coordination to Zn^2+ ^is depicted for the five systems (LuxS_apo-LuxS+2KRI). The active sites are highlighted in the figure with the Zn^2+ ^being depicted as blue van der Waals spheres and the coordinated water molecules are represented as dark pink sticks. The ligand molecules are depicted as dark grey line representation.

All the dynamically stable cliques and communities are evaluated for the five systems under study and the major ones are pictorially depicted in Figure 4 (upper panel). The specific details of the cliques/communities in each of the five systems are presented schematically in Additional file 1: Supplemental Figure SA5(a-c) and Additional file 1: Supplemental Figure SA6(a-b). Our focus is mainly on cliques and communities near the active site, which is at the interface, and the ones that undergo major reorganization on subsequent ligand binding. It can be easily seen from the figures [Figure 4(upper panel), Additional file 1: Supplemental Figure SA5-SA6] that there is an overall increase in the number of cliques and also a marked increase in the aggregation of cliques (both k = 3 and k = 4) to give rise to large communities on going from native LuxS (LuxS_apo) to mono-liganded (LuxS+SRH and LuxS+KRI) and finally to the bi-liganded state (LuxS+2SRH and LuxS+2KRI). This clearly indicates that the system becomes more rigid with subsequent ligand binding. Also the cliques and communities represent the network of amino acid interactions in the different ligand bound states of LuxS.

The disjointed pattern in unliganded LuxS with several small cliques and communities is indicative of the flexibility of the native enzyme, which is proposed to facilitate the initial approach and binding of the ligand. On binding of KRI/SRH to one subunit of LuxS, there is an increase in the overall number of cliques. More complexity is introduced in the network as is indicated by the merging of cliques to give larger communities as compared to the unliganded protein. It is worth noting that there occurs a prominent re-organization in the cliques and community pattern in the vicinity of the active site to which SRH/KRI binds (the catalytic region in one of the subunits) in both the active and inactive form of the enzyme. Also it is interesting to note that the number of cliques and communities at the dimer interface undergoes a slight increment in LuxS+SRH and LuxS+KRI and the rigidity is partially communicated to the other subunit as well, thereby making the whole system partially rigid. The bi-liganded system (LuxS+2SRH and LuxS+2KRI) exhibits a dramatic change in the community configuration, mainly around the catalytic regions and their connecting interface, as compared to the native as well as the mono-liganded state with the emergence of giant communities encapsulating the active sites and the dimer interface. These giant communities increase the rigidity around the active sites and the dimer interface of LuxS. Thus the two subunits are now held in a highly rigid architecture and we propose that the formation of such highly modular structures involving both the subunits of LuxS gives rise to a particular highly populated conformation for LuxS+2SRH/LuxS+2KRI. However, it is worth mentioning that LuxS+2KRI is asymmetric in terms of community pattern around its active sites, one being more rigid and compact than the other [Additional file 1: Supplemental Figure SA6(b)]. This is in contrast to LuxS+2SRH where both the active sites are encapsulated by a single giant community with comparable rigidity around them [Additional file 1: Supplemental Figure SA5(c)].

**Figure 5:**
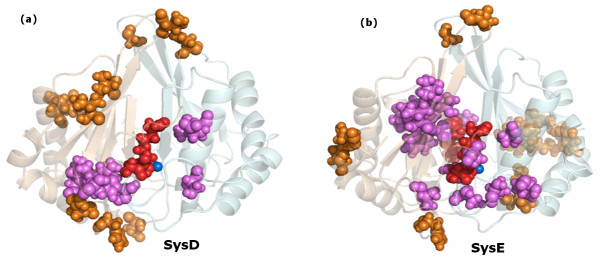
**Unique clique residues for the active ligand bound forms of LuxS**. The unique clique forming residues in (a) LuxS+KRI w.r.t LuxS+SRH, and (b) LuxS+2KRI w.r.t LuxS+2SRH. The two subunits are given in transparent new cartoon representations and are coloured differently. The unique residues are represented as orange van der Waals spheres and the ones which are near the active sites are depicted as violet van der Waals spheres. It is evident that a larger number of unique clique forming residues are near the active sites of LuxS+2KRI.

A comparison of the number of cliques and the participating residues in LuxS+KRI and LuxS+2KRI with their inactive ligand-bound counterparts [LuxS+SRH (mono-liganded) and LuxS+2SRH (bi-liganded)] is presented in Table 2 respectively. Although the two systems are very much similar at the residue participation level during clique formation, they are considerably different at the actual connection level which is obvious from the small number of common cliques between them. This indicates that mostly the same residues are rewired differently in the active and the inactive ligand bound forms of LuxS. However, there are some residues unique to the active forms of LuxS for both the mono and biliganded states with respect to their inactive counterparts and they are pictorially depicted in Figure 5 and listed in Additional file 2: Supplemental Table SB3. It is interesting to note that majority of such unique clique forming residues in the active ligand bound forms of the enzyme are near the active sites and/or the dimer interface [Figure 5, Additional file 2: Supplemental Table SB3]. We propose that such residues provide the additional catalytic framework for the 2-ketone intermediate (KRI) bound (active) forms and thus distinguish the active and inactive ligand bound states in atomistic detail.

**Table 2:** Comparison between the number of cliques and clique forming residues in monoliganded and biliganded states of LuxS

Common cliques	LuxS+SRH	LuxS+KRI	Common cliques	LuxS+2SRH	LuxS+2KRI
**LuxS+SRH**	44	16	**LuxS+2SRH**	59	15

**LuxS+KRI**	16	37	**LuxS+2KRI**	15	60

**Common residues** **in cliques**	**LuxS+SRH**	**LuxS+KRI**	**Common residue ** **in cliques**	**LuxS+2SRH**	**LuxS+2KRI**

**LuxS+SRH**	95	60	**LuxS+2SRH**	90	66

**LuxS+KRI**	60	78	**LuxS+2KRI**	66	95

It is evident from the table that only a small number of common cliques are present between the mono and biliganded states (both active and inactive ligand bound forms for each) of LuxS, whereas the number of common residues constituting the cliques is quite high between them. This is indicative of the fact that majority of the common residues are rewired differently to give rise to different cliques between the active and inactive ligand bound states of the enzyme.

### Hubs

**Figure 6:**
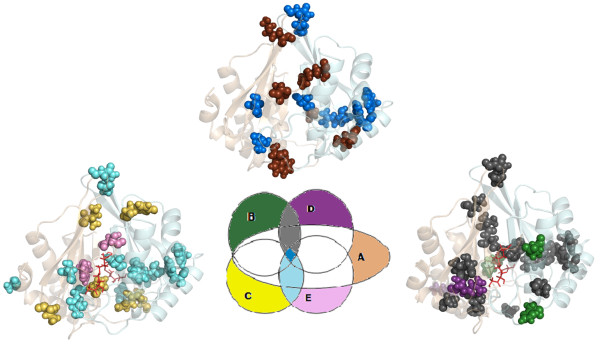
**Comparison between the hub residues in LuxS_apo-LuxS+2KRI**. The central Venn diagram depicts the five systems (LuxS_apo-LuxS+2KRI) as A-E respectively and the different regions are coloured differently. The common regions between LuxS_apo-LuxS+2KRI, LuxS+SRH-LuxS+KRI, and LuxS+2SRH-LuxS+2KRI are coloured deep blue, grey and cyan respectively. The regions which are exclusive to each of LuxS_apo-LuxS+2KRI are highlighted as brown, deep green, yellow, violet, and pink respectively. Three dimensional representations of such comparisons are also given with the two subunits depicted as new cartoon and coloured differently. The common and exclusive hub residues are represented as van der Waals spheres and colour-coded according to the Venn diagram.

Hubs are highly connected amino acid residues (more than three connections) and are known to impart robustness to real world networks [16]. The dynamically stable hubs are identified at I_min _= 2.5%, the same criterion that is chosen for evaluation of cliques. The hubs picked up from our analysis are summarized in Additional file 2: Supplemental Table SB4 and whether the hub residue is a member of a clique is also mentioned in this table. The number of hubs increases to an appreciable extent on going from LuxS+SRH to LuxS+2SRH. On the other hand, on going from LuxS_apo to LuxS+SRH, there is no appreciable increase in the number of hubs. However, it is striking to note that on going from the native (LuxS_apo) to the mono and biliganded active forms (LuxS+KRI and LuxS+2KRI), there is a decrease in the number of hubs. It is to be noted that majority of the hub residues in LuxS_apo-LuxS+2KRI also participate in the cliques and communities recognized for that system. A rigorous comparison between the hub residues for the five systems is pictorially presented in Figure 6 [residuewise details are presented in Additional file 2: Supplemental Table SB5]. It is evident that the hubs that are sacrificed in the active ligand bound forms of LuxS (LuxS+KRI and LuxS+2KRI) are mostly peripheral. It is worth mentioning that many of the common hubs between the active and inactive ligand bound forms of LuxS are near or at the interface.

Hubs are indicative of localized rigidity, whereas extended rigidity in the system is captured by cliques and communities. In the activated ligand bound LuxS states, the extended rigidity is increased at the expense of majorly peripheral localized rigidity with decrease in the number of hubs.

### Water dynamics around Zn^2+^

The dynamics of the water molecules coordinated to Zn^2+ ^in the two subunits of LuxS exhibit interesting variations in different ligand bound states and are pictorially represented in Figure 4 (lower panel). The coordination of water molecules with Zn^2+ ^is followed along the trajectory for each system and a dynamically stable water molecule is one that is present in atleast 25% of the simulation snapshots [a detailed description of the coordinated water molecules including the percentage of their participation along the simulation trajectory in LuxS_apo-LuxS+2KRI are summarized in Table 3 and Additional file 1: Supplemental Figure SA7]. It is evident from Figure 4(a-e) (lower panel) and Table 3 [also Additional file 1: Supplemental Figure SA7] that there is a reduced coordination of water to Zn^2+^, both in terms of number and percentage of participation along the trajectory in LuxS+2SRH and LuxS+2KRI (biliganded forms) to different extents. Such an observation is in good agreement with the fact that LuxS+2SRH and LuxS+2KRI have increased interface compactness in contrast to LuxS_apo, LuxS+SRH and LuxS+KRI [Figure 4(a-e) (upper panel)]. It appears that the compact dimer interface in LuxS+2SRH and LuxS+2KRI contributes to sequester the ligand from the water environment. We have also analyzed the amino acid residues with Cα atoms within a coordination sphere of 4Å from Zn^2+ ^and the ligands (SRH/KRI) in the different complexes under study [the result is summarized as Additional file 2: Supplemental Table SB6 and pictorially depicted as Additional file 1: Supplemental Figure SA8]. These surrounding residues encapsulate the ligands and affect the water/s in the active site. However, as the rigidity around one active site in LuxS+2KRI is comparatively less, it has a greater coordination with water as compared to LuxS+2SRH [Figure 4(c,e)]. This analysis also uncovers the inherent asymmetry in the native enzyme and its differentially liganded forms as the water coordination pattern is noticeably distinct between the two subunits [Figure 4(lower panel), Table 3].

**Table 3:** Variations in the water coordination of Zn^2+ ^in LuxS_apo-LuxS+2KRI

LuxS_apo			LuxS+SRH			LuxS+2SRH			LuxS+KRI			LuxS+2KRI		
6482 WAT	98.71	A	4961 WAT	100	B	6054 WAT	25.02	B	2089WAT	97.51	B	1275WAT	71.70	A
6470 WAT	100	A	7353 WAT	100	B				1186WAT	79.81	A	3375WAT	71.41	A
6453 WAT	100	A	4920 WAT	100	B				4056WAT	100	B	5695WAT	71.21	B
4502 WAT	99.65	B	3067 WAT	42.28	A				4529WAT	100	B	7627WAT	68.44	A
4449 WAT	100	B							4074WAT	76.35	A			

The first column indicates the water molecule that is coordinated to Zn^2+ ^in either subunit, the second column represents the participation (in %) of these water molecules in coordination along the simulation trajectory for the five systems (10 ns each) and the third column summarizes the subunits of LuxS to which the coordination has occurred.

### Conformational Mapping of Essential Dynamical Modes

The essential dynamics analysis (details in Methods section) captures the important dynamical modes of the simulated protein. The plot of RCPF [Additional file 1: Supplemental Figure SA9] indicates that the top two modes accounts for about 35-52% of the overall dynamics for LuxS_apo-LuxS+2KRI. The 'essential plane' constructed from the top two modes represents the major conformational degrees of freedom and the main conformational transitions of the system under study in its various states of ligation.

**Figure 7:**
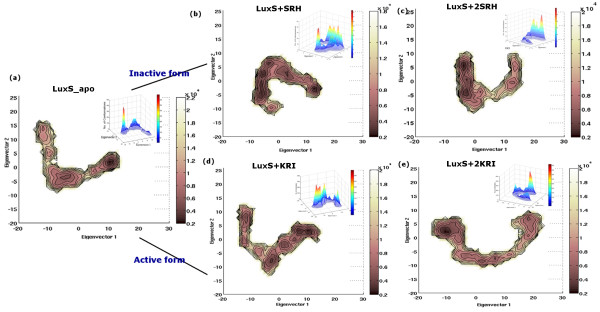
**Helmholtz free energy contour map and population distribution profile**. The Helmholtz free energy contour map for each of the five simulations (on LuxS_apo-LuxS+2KRI, 10 ns each) and the corresponding population distribution in the 'essential plane' are given in inset.

Conformations sampled every 1ps are projected onto the grided 'essential plane' (details described in the Methods section) and 3D-contour maps of population distribution as a function of the position in the essential plane is obtained for the five systems: LuxS_apo-LuxS+2KRI [Figure 7(a-e) inset]. These population distribution profiles essentially represent the most accessible conformational space following ligand binding in proteins as has been established by our analysis of tryptophanyl-tRNA synthetase [23]. We have also extracted the specific snapshots giving rise to a particular peak (peaks indicated as A, B, etc in Additional file 1: Supplemental Figure SA10(a-e)) in the population distribution profile of LuxS and the results are listed in Additional file 2: Supplemental Table SB7.

Our results for the five systems indicate that there is a marked re-distribution of protein conformational ensemble on binding of ligands (both SRH/KRI) to LuxS. For LuxS_apo, the population distribution profile has one global minima contributing to only about 30% of the total conformational ensemble and a very broad peak. On addition of single SRH/KRI to LuxS, the conformational space is spread out and composed of a large number of peaks in contrast to the unliganded LuxS. It is amazing to note that on binding of the second KRI/SRH, the whole system becomes highly rigid and stable as is also indicated by a narrow population distribution profile accounting for about 45-75% of the total ensemble of conformational states. This is indicative of the stability and compactness brought into the system on binding of the second ligand.

### Mapping of 'Essential Conformational Space' with Network Parameters

It is striking to note that the results obtained from the conformational analysis in terms of network parameters such as cliques, communities and hubs are in good agreement with the population distribution profiles. The minimum number of cliques and communities are found in the unliganded LuxS implying maximum flexibility, which is also shown by its population distribution profile [Figure 7(a)]. In LuxS+2SRH and LuxS+2KRI, the number of cliques and communities are maximized with giant community/communities being formed around the active sites. This is also reflected in the narrow population distribution profiles for the bi-liganded forms of LuxS enzyme [Figure 7(c,e)]. However, LuxS+SRH and LuxS+KRI are interesting cases. Here we observe increased rigidity compared to LuxS_apo. Rigidity is induced mainly in the subunit bound to the ligand. Such a conformation appears as though it is an intermediate state and the system has the conformational features of both native and biliganded states [Figure 7]. Although, in general, rigid structures give rise to one major population, these results indicate partial rigidification can give multiple populations.

We have also extracted the specific snapshots from the trajectories giving rise to a particular peak (LuxS_apo-LuxS+2KRI have given rise to 2, 4, 2, 4, and 1 major peak/s as highlighted in Additional file 1: Supplemental Figure SA10) in the population distribution profile. We have further analyzed these peaks in terms of cliques and communities [Additional file 2: Supplemental Table SB8]. These parameters beautifully capture the intricate side-chain variations and thus allow us to compare the conformations within and across simulations in molecular detail, in contrast to the conventional analysis techniques. It is evident that conformations under one peak are distinctly different from that under other peaks within a system. Further, the conformational similarity decreases between peaks from different systems, as measured by the number of common cliques.

### Ligand Induced Changes in the Free Energy Landscape

Using the population distribution profile, Helmholtz free energy is calculated from equation 3[43,44] and the free energy contour maps are plotted [Figure 7(a-e)] for all the five simulations. It is clear that the conformational space accessible to LuxS+SRH and LuxS+KRI (monoliganded forms of LuxS) is much higher with a large number of energetically close conformations than their corresponding biliganded counterparts. This may indicate that the mono-liganded system (LuxS+SRH and LuxS+KRI) is not very stable and thus not capable of catalytic function with efficacy. On the other hand, the rigid and highly modular bi-liganded state may be necessary for the functionality of the enzyme. Each system has an ensemble of conformations in equilibrium with a major conformation/s being dominant. The lowest Helmholtz free energy values for the minima in the free energy landscape for each of the five systems (LuxS_apo-LuxS+2KRI) are 10.439, 13.517, 11.843, 12.894, and 12.850 KJ/mol respectively.

## Discussion

In the Introduction section we have discussed about the various facets and challenges involved in understanding the role of protein conformational changes in enzyme catalysis. It has also been pointed out that allostery can be guided by enthalpic factors and/or entropic factors [3,4]. In order to calculate these contributions from the simulations, a rigorous assessment of the associated conformational changes is a necessity. The simulation trajectory analyses such as the root mean square deviation (RMSD), residue wise RMSD and pairwise correlations between residues provide some information on the conformational fluctuations. However, minor variations at the side-chain level and perturbations due to collective influence of a set of residues are largely elusive from these analyses. Our recent studies on tryptophanyl-tRNA synthetase have shown that the ligand induced changes in the free energy landscape can be efficiently evaluated using the concepts of essential dynamics and network parameters in unison [23]. The essential dynamics analysis enables us to represent the population distribution profile for the protein in different ligand bound states. The network parameters on the other hand enable identification of the amino acid networks that act in coherence with the shift in conformational equilibrium. Thus our method beautifully captures the intricate changes at the molecular level that is associated with the re-distribution of protein conformational states in response to ligand binding. We have applied our methodology to the bacterial quorum sensing protein LuxS from *H.pylori *to probe into the structure and function of this dimeric protein from a molecular perspective. We emphasize the fact that major conformational changes at the backbone level need not necessarily take place during ligand binding to a protein. Rewiring of side chain interactions are often sufficient to bring about functionally relevant changes in the rigidity/flexibility of the protein structure. Our investigation of the five systems (LuxS_apo-LuxS+2KRI) of LuxS and its complexes unravels the relationship between its structure and function. Major reorganisations are observed in the structure of LuxS in its differentially liganded forms as is evident from the analysis of dynamically stable network parameters [Figures 4, 5 and 6, Additional file 2: Supplemental Table SB8]. Below, we have addressed issues like asymmetry and co-operativity between the two subunits, the accessible conformational space, free energy changes and the dynamics of water coordinated to Zn^2+ ^at the active site in both the active and inactive ligand bound states. Ideally longer simulations are required to obtain the full dynamics involving all possible states. However, certain conformational changes, especially at the side chain interaction level, do take place in a timescale of 10 ns simulations (on multiple complexes of LuxS) which provide sufficient data to obtain relative free energies of different accessed states. The rigorous information on free energy changes from existing techniques (like MM-PBSA and Umbrella sampling) is more expensive and here we propose an alternative and computationally less expensive method to obtain information on free energy changes subsequent to ligand binding. Based on these investigations, we have finally probed the mechanistic aspects of catalysis.

The asymmetry of this dimeric protein is evident at various levels such as the hydrogen bonding pattern between protein and ligand, the participation of amino acid residues at the interface, inequality in rigidity at the active sites as measured from different number of cliques in the two subunits [Table 4]. It is also reflected in the water coordination to the Zn^2+ ^at the active sites. The asymmetry is maximally enhanced in the complex bound to two molecules of KRI (LuxS+2KRI). It is interesting to note the differences in the network parameters [Figure 5,6] between the active and the inactive ligand bound complexes (a residue-wise summary is presented in Additional file 2: Supplemental Tables SB3, SB5). Furthermore, we see that although some residues forming the cliques are common to both the active and inactive ligand bound complexes, some are unique to a particular ligand bound state. At the structural level, most of these unique residues are near the active site/interface and some of them have been experimentally shown to affect the activity [Additional file 2: Supplemental Table SB3] [45]. These unique residues contribute majorly to the subtle conformational differences between the active and inactive ligand bound forms of the enzyme and have been pictorially depicted in Figure 5. Similarly, we have also identified the hub residues unique to the active ligand bound forms of LuxS [Figure 6, Additional file 2: Supplemental Table SB5]. It is striking to note that such atomic level insight into ligand induced conformational changes can be provided only at the detailed side-chain interaction level (using network parameters) and remains largely elusive to conventional methods of structural analyses (like RMSD, residue-wise RMSD). The cooperativity between the two subunits as seen from dynamic cross-correlation (DCCM) and network parameters also differ in different liganded states. Further, considering the results from all the systems, we have also listed some of the residues that contribute to the rigidity of the structure (as hubs or part of cliques presented in Additional file 2: Supplemental Table SB4). Some of the conserved hub residues participating in cliques (like 5 S, 10 H, 38R, 54E, etc) have been previously mutated to show decreased enzyme activity. We further predict residues like 6F, 36 D, 39F, 53L, 62R, 80Y, and 108V as experimental targets for mutations based on their contribution in maintaining the rigidity of the enzyme framework as parts of hubs/cliques and conservation along a large number (30 sequences) of LuxS sequences.

**Table 4:** Distribution of cliques in the core (subunit A/B) and the dimer interface for LuxS_apo-LuxS+2KRI

LuxS_apo (k = 3)	LuxS+SRH (k = 3)	LuxS+2SRH		LuxS+KRI (k = 3)	LuxS+2KRI (k = 3)
				
		(k = 3)	(k = 4)		
Subunit A: 14	Subunit A: 21	Subunit A: 15	Subunit A: No cliques	Subunit A: 8	Subunit A: 19
Subunit B: 14	Subunit B: 11	Subunit B: 13	Subunit B: 1	Subunit B:15	Subunit B: 17
Interface: 7	Interface: 12	Interface: 27	Interface: 3	Interface: 14	Interface: 24

The present study has enabled us to track the ligand induced changes in the protein conformation, redistribution of the populations and associated free energy changes effectively through structure network representation. The Helmholtz free energy contour maps for the five systems portray the ligand induced changes in the free energy landscape for LuxS. Upon ligand binding, the proteins may undergo conformational changes and/or changes in the distribution of their population. Here we are able to obtain changes in the atomic description of conformations and their populations in equilibrium as a function of the bound ligand. Such an investigation provides a tool to capture the subtle conformational changes in proteins in response to ligand binding in a detailed and quantitative manner.

**Figure 8:**
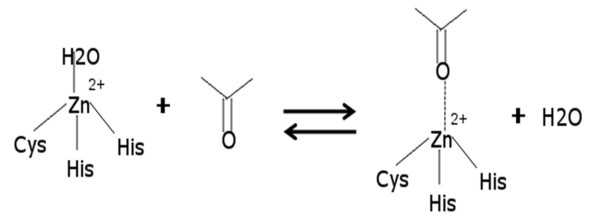
**Reversible first step in the reaction between SRH and LuxS**. Reversible exchange of the water molecule occupying the fourth coordination site of Zn^2+ ^with the carbonyl group formed upon rearrangement of S-ribosylhomocysteine.

In order to find the biological relevance of our observations we delve into the LuxS catalytic mechanism. In the recent past, the mechanism of the LuxS enzyme action has been investigated in detail [28,46-48]. A detailed mechanism for LuxS catalysis has been proposed based on NMR studies, mass spectrometric analysis and kinectic experiments by Pei's group. The proposed mechanism of LuxS action from these studies is schematically described in Additional file 1: Supplemental Figure SA11. One step in the catalytic reaction of LuxS is a reversible exchange of the water molecule occupying the fourth co-ordination site of zinc (the other three sites coordinate with two His and one Cys) in the active site with a carbonyl group in S-ribosylhomocysteine (open form) as shown schematically in Figure 8[28,45]. As mentioned above, we observe that in active biliganded (LuxS+2KRI) form of the enzyme, the asymmetry is marked with one of the active sites being encapsulated by a giant community with more hubs [Additional file 1: Supplemental Figure SA6(b)], greater number of ligand-protein dynamically stable hydrogen bonds, lower percentage of Zn^2+^-water coordination along the trajectory and so on as compared to the other. We find that binding of ligands to both the active sites results in a strongly compact interface and increase the rigidity at the active sites. This feature is advantageous to exclude water from the active site, which in turn facilitates the forward reaction as shown schematically in Figure 8 and thus the catalytic cleavage of S-ribosylhomocysteine. However, this rigidity and exclusion of the water in the active biliganded complex (LuxS+2KRI) is asymmetric. This leads us to speculate 'half-of-the-sites-reactivity' which means that only one of the sites is catalytically active while the ligand is bound to both the active sites. It would be interesting to verify this proposal by experimental studies such as differential binding constants and catalytic activities of the mono and bi-liganded states. Thus, the mechanism of LuxS action is investigated in light of our observations to elucidate the optimum structural conditions for LuxS catalysis.

## Conclusions

In summary, we have offered a novel generalized method to visualize the major conformational space associated with a protein and its various ligand bound states and simultaneously explore the molecular detail of the associated conformational transitions in terms of the dynamically stable network parameters and Helmholtz free energy changes. We have applied the method to *H.pylori *LuxS and its different ligand bound states to investigate the structure-function relationship. The study uncovers some of the structural events that lead to an efficient functional outcome (i.e, the catalytic action) in LuxS. Here we are able to capture the ligand induced conformational changes which includes the side-chain re-orientations and re-distribution of conformational populations. We are also able to identify the salient features resulting from the binding of the active (KRI) and the inactive (SRH) forms of S-ribosylhomocysteine to *H.pylori *LuxS. Thus our analysis of LuxS has illuminated upon the catalytic mechanism of action of the enzyme. Our analyses also provided a generalized tool to capture the subtle ligand induced conformational changes in any protein of known structure.

## Abbreviations

LuxS: S-Ribosylhomocysteine lyase; PSN: Protein Structure Network; SRH: S-Ribosylhomocysteine (ribose form); KRI: catalytic 2-ketone intermediate; DCCM: Dynamic Cross-correlation map; DPD: 4,5-dihydroxy-2,3-pentanedione.

## Authors' contributions

MB and SV designed the study, MB performed the calculations, MB and SV analyzed the data, MB and SV drafted the manuscript. All authors read and approved the final manuscript.

## Supplementary Material

Additional file 1 **Supplementary Figures**. The file contains supplementary figures (Figure SA1 to Figure SA11) in pdf format. Click here for file

Additional file 2 **Supplementary Tables**. The file contains eight supplementary tables (Table SB1 to Table SB8) in pdf format. Click here for file
