# Supplementary material for: Elucidation of the conformational free energy landscape in H.pylori LuxS and its implications to catalysis
Source: BMC Struct Biol. 2010 Aug 12;10:27. doi: 10.1186/1472-6807-10-27 (PMC2929236; doi:10.1186/1472-6807-10-27)
Supplement: Additional file 1 — Supplementary Figures. The file contains supplementary figures (Figure SA1 to Figure SA11) in pdf format. [file 1472-6807-10-27-S1.PDF]

Supplemetary Figures

Figure SA1

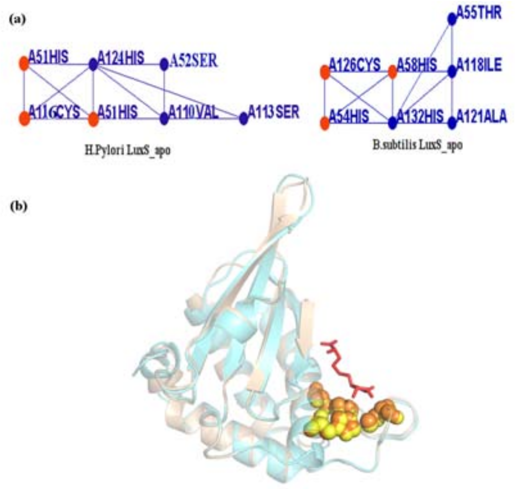

Side chain orientations around the active sites of *B.subtilis* and *H.pylori* LuxS. (a) The communities involving the active site residues are depicted. Residues participating in these communities are same/similar in the two species. The ligand binding residues are highlighted by red circles. (b) Pictorial depiction of the aligned structures of LuxS from *H.pylori* and *B.subtilis* in cartoon representation. The active site and ligand binding residues (51H, 55H, and 116C) are highlighted as van der Waals' spheres and colored yellow for *B.subtilis* and orange for *H.pylori* LuxS. The ligand (SRH) is shown as red sticks. The figure clearly depicts the identity of the binding pocket in LuxS from the two species.

Figure SA2

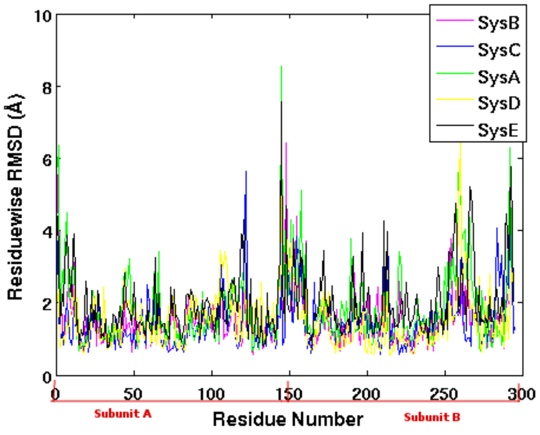

Average residue-wise RMSD profiles for SysA-E (LuxS_apo, LuxS+SRH, LuxS+2SRH, LuxS+KRI and LuxS+2KRI respectively) over the simulation trajectories (10 ns each). The

residues from 1-145 belong to subunit A and those from 147-295 belong to subunit B of this dimeric protein.

Figure SA3

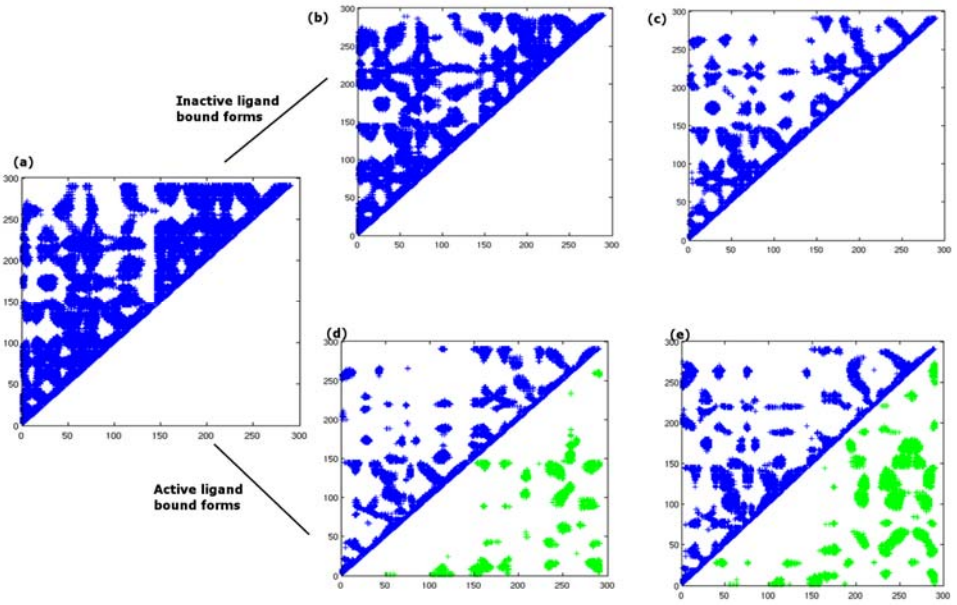

Dynamic cross correlation map (DCCM) representing the collective atom fluctuations for (a) LuxS_apo, (b) LuxS+SRH, (c) LuxS+2SRH, (d) LuxS+KRI, and (e) LuxS+2KRI respectively. Strong positive correlation [Cij = ±(0.8-1.0)] and moderate anti-correlation [Cij = ±(0.5-1.0)] are shown in the figure. The lower and upper halves of the triangle represent negative and positive correlations respectively. Regions between X-axis: 0-145, Y-axis: 0-145 and X-axis: 147-295, Y-axis: 147-295 indicate correlated fluctuations within a subunit and that between X-axis: 0-145, Y-axis: 147-295 and X-axis: 147-295, Y-axis: 0-145 indicate cross-correlation between the two subunits.

Figure SA4

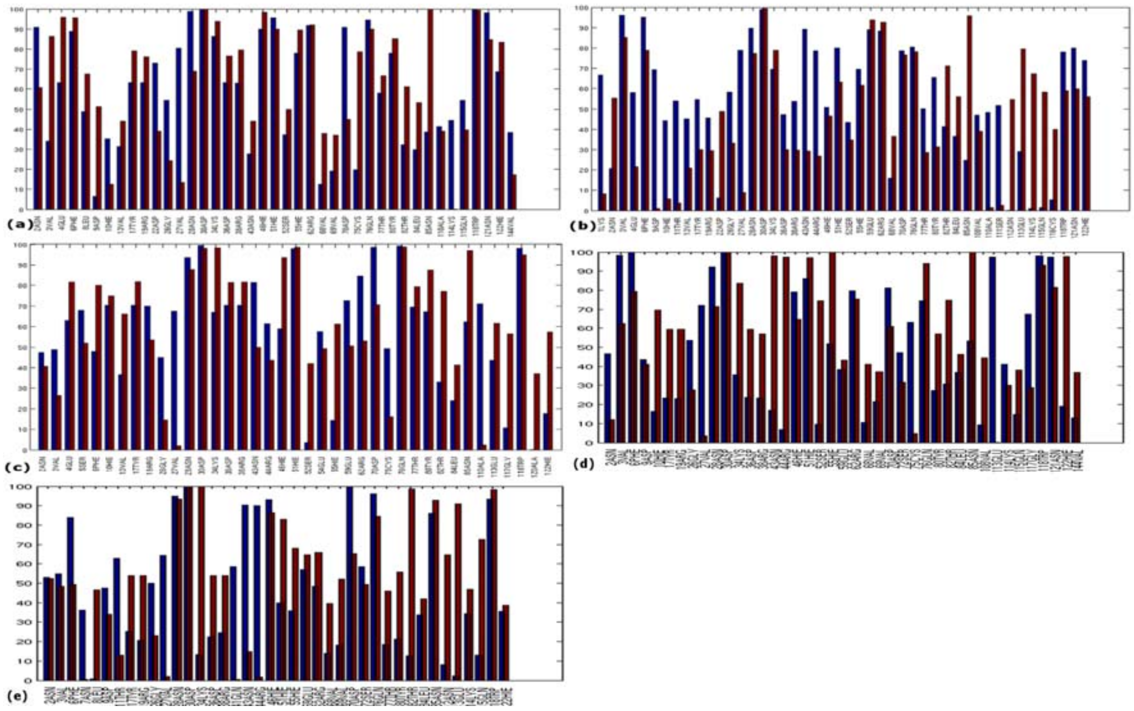

Results of interface dynamics analysis represented in the form of bar graphs; blue and red bars indicating same residues from the two different subunits of LuxS for (a) LuxS_apo, (b) LuxS+SRH, (c) LuxS+2SRH, (d) LuxS+KRI, and (e) LuxS+2KRI respectively. The height of the bar indicates the % of occurrence of that residue in the interface during the simulation. The residue numbers of the dynamically stable interface residues are given along the x-axis. The asymmetry of participation of the residues to the dimer interface is evident from the figure.

Figure SA5(a-c)

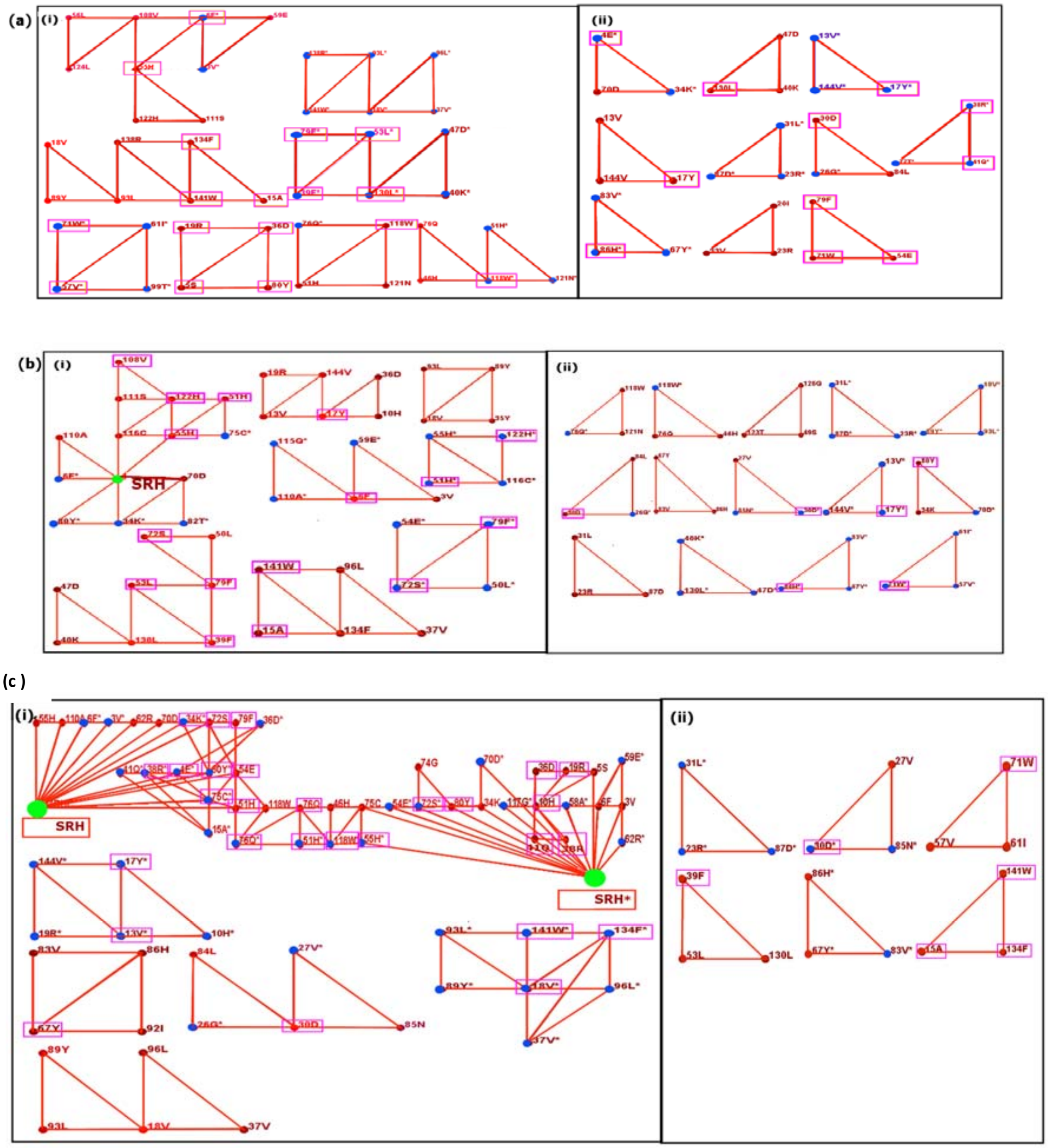

The (i) communities and (ii) cliques represented for (a) LuxS_apo, (b) LuxS+SRH, and (c) LuxS+2SRH respectively. The residues coming from chain A and chain B are indicated with a red/maroon and blue node respectively and the residues from chain B are marked with a '*'. The ligands are highlighted by green circles. The hubs participating in the cliques and communities are indicated by pink rectangles.

Figure SA6(a-b)

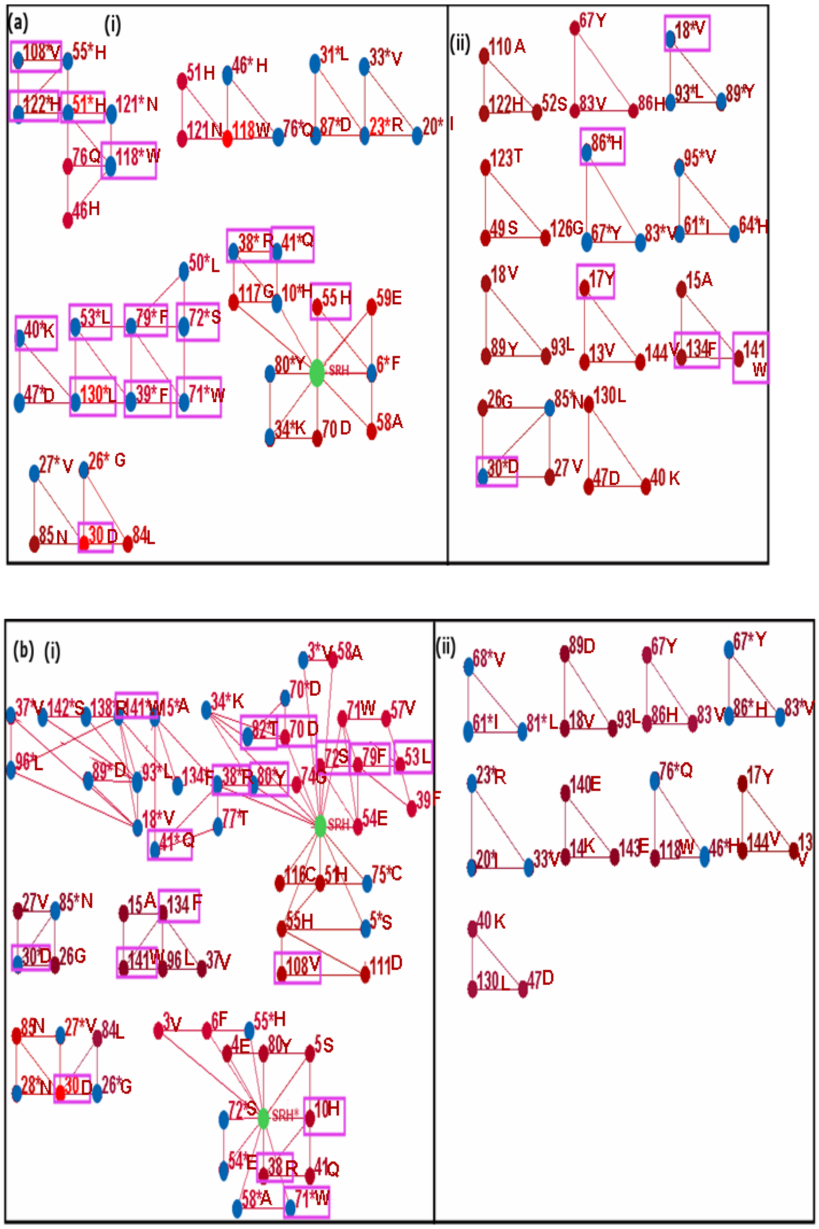

The (i) communities and (ii) cliques represented for (a) LuxS+KRI, and (b) LuxS+2KRI respectively. The residues coming from chain A and chain B are indicated with a red/maroon and blue node respectively and the residues from chain B are marked with a '*'. The ligands are highlighted by green circles. The hubs participating in the cliques and communities are indicated by pink rectangles.

Figure SA7

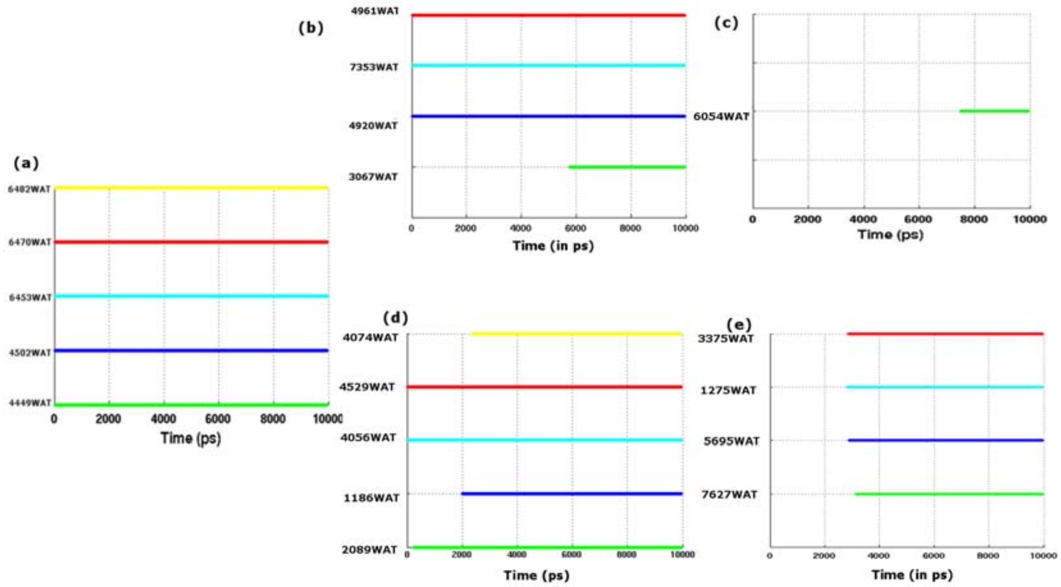

The number and percentage of participation of water molecules (along y-axis) around Zn^{2+} in the two subunits of (a) LuxS_apo, (b) LuxS+SRH, (c) LuxS+2SRH, (d) LuxS+KRI, and (e) LuxS+2KRI respectively along the simulation trajectory.

Figure SA8

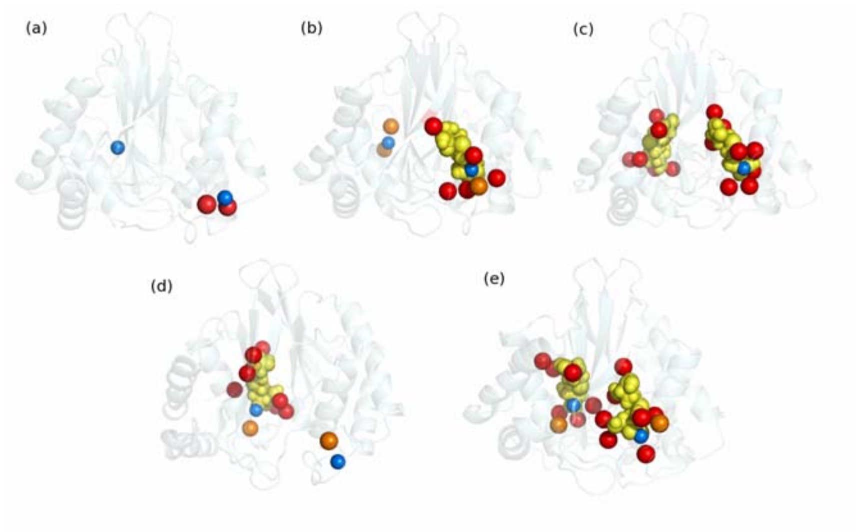

Amino acid residues with Cα within 4Å of Zn^{2+} and ligands (SRH/KRI) in the different ligand bound forms of LuxS [LuxS_apo-LuxS+2KRI in (a-e)] respectively. The protein backbone is represented as transparent new cartoon. The Zn^{2+} and ligands are depicted as

blue and yellow spheres respectively. The Cα of residues within 4Å of Zn^{2+} and ligands are represented as orange and red spheres respectively.

Figure SA9

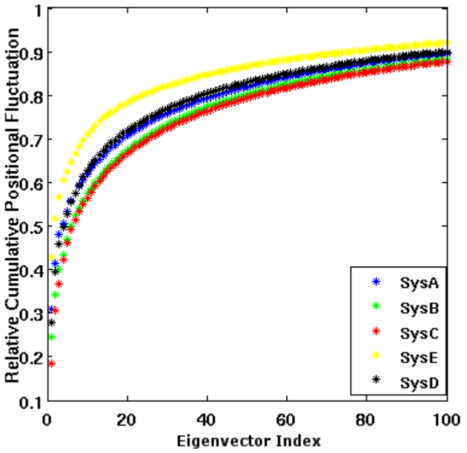

Relative cumulative positional fluctuations (RCPF) for LuxS_apo-LuxS+2KRI.

Figure SA10

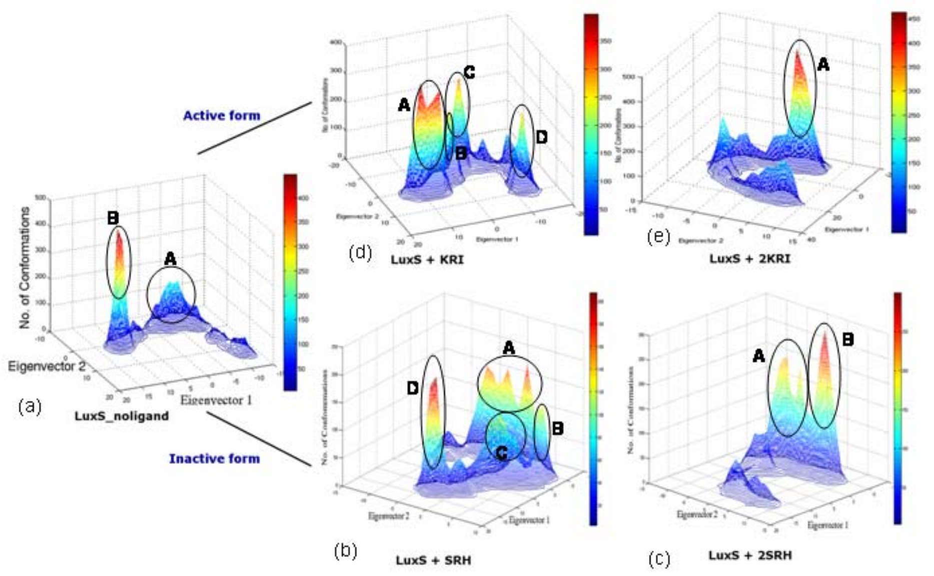

The population distribution profiles for (a) LuxS_apo, (b) LuxS+SRH, (c) LuxS+2SRH, (d) LuxS+KRI, and (e) LuxS+2KRI in the ‘essential plane’ defined by the top two eigenvalues. The major peaks are indicated in the Figure SAs A, B etc.

Figure SA11

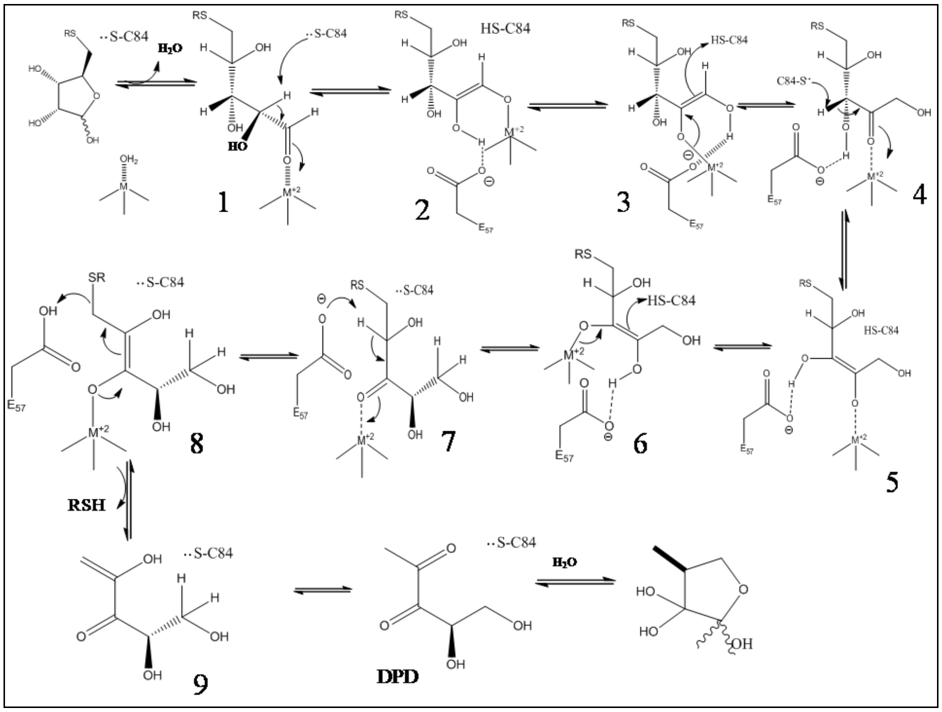

Schematic representation of LuxS enzyme action
