# Supplementary material for: Elucidation of the conformational free energy landscape in H.pylori LuxS and its implications to catalysis
Source: BMC Struct Biol. 2010 Aug 12;10:27. doi: 10.1186/1472-6807-10-27 (PMC2929236; doi:10.1186/1472-6807-10-27)
Supplement: Additional file 2 — Supplementary Tables. The file contains eight supplementary tables (Table SB1 to Table SB8) in pdf format. [file 1472-6807-10-27-S2.PDF]

Supplementary Tables

Table SB1: Dynamically stable hydrogen bonds between KRI and LuxS

(a)

DONOR ----> ACCEPTOR	% of occurence
62R(NH1) - **KRI**(N1)	59.64
117G(N) – **KRI**(O3)	74.68
**KRI** (N1) – 70D (OD1)	92.52
**KRI**(N1) – 71W(O)	94.12

(b)

DONOR ----> ACCEPTOR	% of occurence	DONOR ----> ACCEPTOR	% of occurence
62R(NH1) - **KRI**(O6)	97.83	62R*(NH1) – **KRI***(O6)	97.40
62R(NH1) - **KRI**(O2)	58.18	62R*(NH2) – **KRI***(O6)	89.22
62R(NH2) - **KRI**(O6)	89.35	62R*(NH2) – **KRI***(O2)	62.51
117 G(N) - **KRI**(O3)	59.89	71W*(N) – **KRI*(**O6)	72.15
**KRI**(O1) - 115Q(O)	72.69	**KRI***(N1) - 70D*(OD1)	78.14
**KRI**(O4) - 221G(O)	75.88	**KRI***(N1) - 71W*(O)	98.00
**KRI**(O5) - 152S(OG)	90.57	**KRI***(O2) - 4E(OE2)	62.88
**KRI** (N1) – 70D(OD1)	83.60		
**KRI**(N1) - 71W(O)	99.09		
**KRI** (O2) - 151E(OE1)	99.97		
181K(NZ) - **KRI** (N1)	61.10		
185R(NH1) – **KRI(**O1)	74.02		
185R(NH2) - **KRI(**O1)	72.53		

Dynamically stable hydrogen bonds (present in ≥ 50% of the snapshots) made by the ligand KRI with LuxS residues for (a) LuxS+KRI and (b) the two subunits of LuxS+2KRI. A * after the residue indicate that it has come from subunit B. The adjacent columns indicate the presence of the H-bonds dynamically along the trajectory (in %).

Table SB2: Dynamically stable hydrogen bonds between SRH and LuxS

(a)

DONOR ----> ACCEPTOR	% of occurence
**147SRH**(O6) - 110A(O)	88.21
**147SRH**(O5) - 55H(ND1)	54.15
34K*(LZ) - **147SRH**(N1)	60.32

(b)

DONOR ----> ACCEPTOR	% of occurence	DONOR ----> ACCEPTOR	% of occurence
62R(NH1) - **147SRH**(N1)	96.02	62R*(NH2) - **148SRH***(N1)	99.74
62R(NH2) - **147SRH**(N1)	53.56	71W*(N) - **148SRH***(N1)	57.12
**147SRH**(N1) - 70D(OD1)	81.24	**148SRH***(N1) - 70D*(OD1)	63.67
**147SRH**(N1) - 71W(O)	98.54	**148SRH***(N1) - 71W*(O)	92.46
**147SRH**(O6) - 110A(O)	99.25	**148SRH***(N1) - 72S*(OG)	63.32
**147SRH**(O4) - 4E*(OE2)	79.81	**148SRH***(O5) - 55H*(ND1)	53.13
4E*(N) - **147SRH**(O3)	93.24	**148SRH***(O4) - 10H(ND1)	97.82

Dynamically stable hydrogen bonds (present in $\geq$ 50% of the snapshots) made by the ligand SRH with LuxS residues for (a) LuxS+SRH and (b) the two subunits of LuxS+2SRH. A * after the residue indicate that it has come from subunit B. The adjacent columns indicate the presence of the H-bonds dynamically along the trajectory (in %).

Table SB3: List of unique clique forming residues in mono and bi-liganded active forms (LuxS+KRI and LuxS+2KRI) w.r.t their inactive counterparts (LuxS+SRH and LuxS+2SRH)

**Unique residues in LuxS+KRI cliques w.r.t LuxS+SRH**	**Unique residues in LuxS+2KRI cliques w.r.t LuxS+2SRH**
52S 46H* 121N* 59E 20I* 33V* 27V* 85N 58A 26G 64H* 95V* *10H** 117G *38R** 41Q 53L* 39F*	116C 46H* 13V 144V 17Y *40K* 47D 138R* 142S* 140E 14K 143E 58A 74G* 147V* 71W* 20I* 33V* 108V 111D *5S** 77T* 4E 26G 28N* 81L* 61I* 68V* 82T*

The unique residues which are near the active site are given in bold and the ones which have been mutated are highlighted in italics.

Table SB4: List of Dynamically Stable Hubs (Imin = 2.5%) in LuxS_apo-LuxS+2KRI

RESIDUE	LuxS_apo	LuxS+SRH	LuxS+2SRH	LuxS+KRI	LuxS+2KRI	Conservation
**5S**	+ +	-	-	-	-	#
6F	-	+ +	-	-	-	#
**10H**	-	-	+ +	-	++	#
15A	+ +	+ +	+ +	-	-	
17Y	+ +	+ +	-	++	-	
19R	+ +	-	+ +	+-	-	
30D	+ +	+ +	+ +	++	++	
36D	+ +	-	+ +	-	-	#
**38R**	+ -	+ -	+ +	+-	++	#
39F	-	+ +	+ +	-	-	:
41Q	+ -	-	+ +	-	-	
43N	-	-	+ -	-	-	#
51H	-	+ +	+ +	-	-	#
53L	+ -	+ +	-	-	++	:
**54E**	+ +	-	+ +	-	-	#
55H	+ +	+ +	-	-	-	#
59E	-	+ -	-	-	-	
62R	+ -	-	-	-	-	#
67Y	-	-	+ +	-	-	
70D	-	-	-	-	++	
71W	+ +	-	+ +	+-	-	
72S	+ -	+ +	+ +	+-	++	.

76Q	-	-	+ +	-	-	
79F	+ +	+ +	+ +	-	++	
80Y	+ +	+ +	+ +	+-	-	:
108V	-	+ +	-	+-	++	:
118W	+ +	-	-	-	-	
122H	-	+ +	-	-	-	#
130L	+ +	-	-	-	-	
134F	+ +	+ +	+ +	++	++	
141W	+ +	+ +	+ +	++	++	
4E*	+ -	-	+ +	-	-	
6F*	+ -	-	-	-	-	#
13V*	-	-	+ +	-	-	:
17Y*	+ -	+ +	+ +	+-	-	
18V*	-	-	+ +	++	-	
30D*	+ -	+ +	+ +	++	++	
34K*	-	-	+ +	-	-	
**38R***	+ -	+ -	+ +	++	++	#
39F*	+ -	+ -	-	++	-	:
40K*	-	-	-	++	-	
41Q*	+ -	-	-	++	++	
51H*	-	+ +	+ +	++	-	#
53L*	+ -	-	-	++	+-	:
55H*	-	-	+ +	++	-	#
27V*	+ -	-	-	-	-	
71W*	+ -	+ +	-	++	++	
72S*	-	+ +	+ +	++	-	
**75C***	-	-	+ +	-	-	#
76Q*	-	-	+ +	-	-	

79F*	+ -	+ +	+ -	++	+-	
80Y*	-	-	+ +	-	++	:
82T*	-	-	-	-	++	
86H*	-	+ +	-	++	-	
108V*	-	-	-	++	-	:
118W*	+ -	-	+ +	++	+-	
122H*	+ -	+ +	-	++	-	#
130L*	+ -	-	-	++	+-	
134F*	+ -	-	+ +	+-	-	
141W*	-	-	+ +	-	++	

A '+/-' sign in the first column indicates whether the residue is a/not a hub in that particular system. Similarly, a '+/-' sign in the second column indicates whether hub the residue is present/absent from the cliques for that particular structure respectively. The residues given in bold have been experimentally mutated *(1)*. * after a residues indicates that it has come from subunit B. The last column contains conservation information derived from 30 non-redundant LuxS sequences. #/: means complete/partial conservation, . means semi-conservative mutation.

Table SB5: Hub comparison table for LuxS_apo-LuxS+2KRI

Hubs (2.5%)	LuxS_apo	LuxS+SRH	*LuxS+KRI*	LuxS+2SRH	*LuxS+2KRI*
Invariant ***(8)*** **(Common $^{LuxS\_apo-LuxS+2KRI}$)-SetI**		30D, 38R, 72S, 134F, 141W, 30D*, 38R*, 79F*			
Common to all liganded complexes ***(8)*** **(Common $^{LuxS+SRH-LuxS+2KRI}$)-SetI**	--	30D, 38R, 72S, 134F, 141W, 30D*, 38R*, 79F*			
Common between inactive and active form of mono-liganded LuxS ***(18)*** **(Common$^{LuxS+SRH-LuxS+KRI}$)-SetI+Seta$_1$**	--	17Y, **30D, 38R, 72S**, 80Y, 108V, 134F, 141W, 17Y*, 30D*, 38R*, 39F*, 51H*, 71W*, 72S*, 79F*, 86H*, 122H*		--	--
Common between inactive and active form of bi-liganded LuxS ***(13)*** **(Common$^{LuxS+2SRH-LuxS+2KRI}$)-SetI+Seta$_2$**	--	--	--	10H, 30D, 38R, 72S, 79F, 134F, 141W, 30D*, 38R*,	

				79F*, 80Y*, 118W*, 141W*	
**Exclusive-SetII-VI**	5S, 62R, 118W, 130L, 6F*, 27V*	6F, 59E, 122H	40K*, 108V*	43N, 67Y, 76Q, 13V*, 34K*, 75C*, 76Q*	70D, 82T*

The combinations of systems (LuxS_apo-LuxS+2KRI) used for the comparison are shown with the corresponding subscripts.

Table SB6: List of amino acid residues with Cα atom within 4Å of Zn^{2+} and ligands (SRH/KRI) in LuxS_apo-LuxS+2KRI

**LuxS_apo**	**LuxS+SRH**	**LuxS+2SRH**	**LuxS+KRI**	**LuxS+2KRI**
Zn^A: ----<br><br>Zn^B: 111S* 115Q*	Zn^A: 116C 5S* 115Q<br><br>SRH^A: 21A* 117G 110A 116C 5S*<br><br>Zn^B: 5S 115Q*<br><br>SRH^B: ----	Zn^A: 5S<br><br>SRH^A: 117G 110A 3V* 5S* 71W 55H 116C 4E*<br><br>Zn^B: 116C<br><br>SRH^B: 117G* 70D* 116C* 72S* 6F 5S 71W* 55H*	Zn^A: 51H<br><br>KRI^A: 117G 74G* 71W 55H 70D 75C* 5S*<br><br>Zn^B: 111S*<br><br>KRI^B: ----	Zn^A: 51H<br><br>KRI^A: 117G 3V* 5S* 74G* 71W 116C<br><br>Zn^B: 6F 116C*<br><br>KRI^B: 70D* 74G 116C* 72S* 5S 71W* 55H* 75C

The amino acid residues with Cα within 4Å of Zn^{2+} and ligands in Subunit A/B of LuxS. Asymmetry is evident between the two subunits. These residues participate in ligand and metal binding and thus affect the water/s in the active site.

Table SB7: Details of the Population distribution profile in Figure 8(a-c).

**LuxS_apo**	**Major Simulation Time interval(ns)**	**Percentage of snapshots under the peak**
Peak A	1-6	45
Peak B	6-10	33
**LuxS+SRH**		
Peak A	0-5	40
Peak B	4-6	5
Peak C	6-9	20.5
Peak D	8-10	12

**LuxS+2SRH**		
Peak A	1-7	48
Peak B	7-10	23
**LuxS+KRI**		
Peak A	6-9	32
Peak B	4-5	14
Peak C	2-4	21
Peak D	0-1, 9-10	12
**LuxS+2KRI**		
Peak A	6-10	42

Table SB8: Clique Comparision Table (inter and intra simulation) for 50% dynamically stable cliques.

Systems	^{SysA}PeakA	^{SysA}PeakB	^{SysB}PeakA	^{SysB}PeakB	^{SysB}PeakC	^{SysB}PeakD	^{SysC}PeakA	^{SysC}PeakB	^{SysD}PeakA	^{SysD}PeakB	^{SysD}PeakC	^{SysD}PeakD	^{SysE}PeakA
^{SysA}PeakA	**26**	3	3	3	3	4	2	2	2	2	0	0	1
^{SysA}PeakB		**7**	1	1	1	2	1	1	2	1	0	0	2
^{SysB}PeakA			**31**	22	24	22	8	9	3	3	0	1	3
^{SysB}PeakB				**34**	26	21	9	9	3	3	0	1	4
^{SysB}PeakC					**43**	31	12	14	4	5	0	1	7
^{SysB}PeakD						**45**	11	12	4	5	0	1	8
^{SysC}PeakA							**36**	26	2	1	0	1	5
^{SysC}PeakB								**40**	2	2	0	1	4
^{SysD}PeakA									**27**	18	0	2	2
^{SysD}PeakB										**27**	0	2	2
^{SysD}PeakC											**0**	2	0
^{SysD}PeakD												**3**	1
^{SysE}PeakA													**31**

The Table clearly indicates relatively more similarity among the intra simulation peaks as compared to the inter simulation peaks in terms of cliques. The cliques for individual peaks are given in bold [the cliques for individual peaks are obtained without including the ligands for simplicity] and the number of common cliques between two peaks is given in normal font. SysA-E represents LuxS_apo, LuxS+SRH, LuxS+2SRH, LuxS+KRI and LuxS+2KRI respectively.
